# Bis{2-[1-(benzyl­imino)­eth­yl]phenolato}palladium(II)

**DOI:** 10.1107/S1600536810031375

**Published:** 2010-08-18

**Authors:** Amalina Mohd Tajuddin, Hadariah Bahron, Wan Nazihah Wan Ibrahim, Bohari M. Yamin

**Affiliations:** a Department of Chemistry, Faculty of Applied Sciences, Universiti Teknologi MARA, 40450 Shah Alam, Selangor, Malaysia; b School of Chemical Sciences and Food Technology, Universiti Kebangsaan Malaysia, UKM 43600 Bangi Selangor, Malaysia

## Abstract

In the title compound, [Pd(C_15_H_14_NO)_2_], the Pd atom lies on an inversion center and is coordinated by two ligand mol­ecules through the O and N atoms in a bidentate manner, forming a slightly distorted square-planar geometry. The dihedral angle between the two benzene rings in the ligand is 76.53 (19)°. The mol­ecular packing is stablized by C—H⋯O and C—H⋯π inter­actions.

## Related literature

For the catalytic activity of palladium(II)–Schiff base complexes, see: Gupta & Sutar (2008 ▶); Lai *et al.* (2005 ▶) and for their anti­tumor activity, see: Garoufis *et al.* (2008 ▶). For related structures, see: Adrian *et al.* (2008 ▶); Wan Nazihah Wan Ibrahim *et al.* (2008 ▶); Chen & Xia (2009 ▶). For bond-length data, see: Allen *et al.* (2002 ▶).
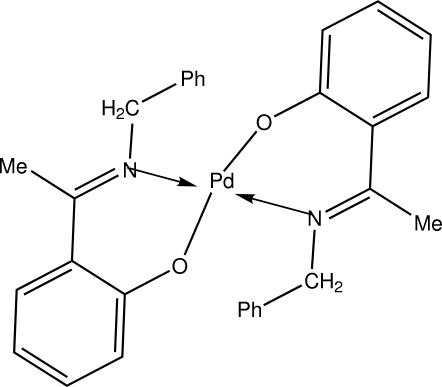


## Experimental

### 

#### Crystal data

[Pd(C_15_H_14_NO)_2_]
*M* *_r_* = 554.94
Monoclinic, 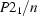

*a* = 11.188 (2) Å
*b* = 9.4460 (17) Å
*c* = 11.984 (2) Å
β = 110.558 (4)°
*V* = 1185.8 (4) Å^3^
*Z* = 2
Mo *K*α radiation
μ = 0.81 mm^−1^
*T* = 298 K
0.28 × 0.20 × 0.12 mm

#### Data collection

Bruker SMART APEX CCD area-detector diffractometer
Absorption correction: multi-scan (*SADABS*; Bruker, 2000 ▶) *T*_min_ = 0.804, *T*_max_ = 0.908
6420 measured reflections
2177 independent reflections
1939 reflections with *I* > 2σ(*I*)
*R*_int_ = 0.038

#### Refinement

*R*[*F* ^2^ > 2σ(*F* ^2^)] = 0.036
*wR*(*F* ^2^) = 0.109
*S* = 1.18
2177 reflections
161 parameters
H-atom parameters constrained
Δρ_max_ = 0.88 e Å^−3^
Δρ_min_ = −0.98 e Å^−3^

### 

Data collection: *SMART* (Bruker, 2000 ▶); cell refinement: *SAINT* (Bruker, 2000 ▶); data reduction: *SAINT* (Bruker, 2000 ▶); program(s) used to solve structure: *SHELXS97* (Sheldrick, 2008 ▶); program(s) used to refine structure: *SHELXL97* (Sheldrick, 2008 ▶); molecular graphics: *SHELXTL* (Sheldrick, 2008 ▶); software used to prepare material for publication: *SHELXTL* (Sheldrick, 2008 ▶), *PARST* (Nardelli, 1995 ▶) and *PLATON* (Spek, 2009 ▶).

## Supplementary Material

Crystal structure: contains datablocks global, I. DOI: 10.1107/S1600536810031375/pv2311sup1.cif

Structure factors: contains datablocks I. DOI: 10.1107/S1600536810031375/pv2311Isup2.hkl

Additional supplementary materials:  crystallographic information; 3D view; checkCIF report

## Figures and Tables

**Table 1:** Hydrogen-bond geometry (Å, °) *Cg*3 and *Cg*4 are the centroids of the C1–C6 and C10–C16 rings, respectively.

*D*—H⋯*A*	*D*—H	H⋯*A*	*D*⋯*A*	*D*—H⋯*A*
C9—H9*A*⋯O1^i^	0.97	2.18	2.862 (4)	127
C8—H8*B*⋯*Cg*4^ii^	0.96	2.57	3.523 (5)	172
C13—H13*A*⋯*Cg*3^iii^	0.93	2.87	3.626 (5)	139

Symmetry codes: (i) 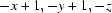; (ii) 
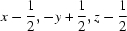
; (iii) 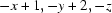.

**Fig. 1.**
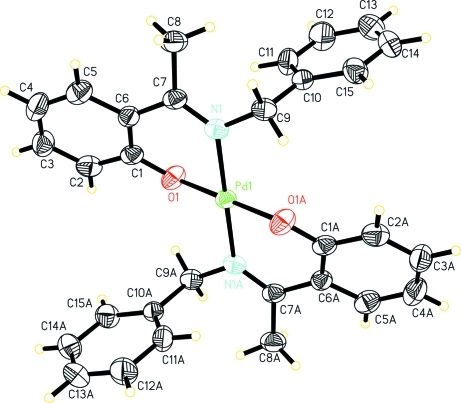
The molecular structure of the title complex with displacement ellipsods drawn at the 50% probability level.

## supplementary crystallographic information

### Comment

The palladium- Schiff bases complexes have found various applications expecially as catalyst (Gupta & Sutar, 2008; Lai *et al.*, 2005) and antitumors activity (Garoufis *et al.*, 2008). The title compound is analogous to the previously reported complex, {2,2'-[(2,2-dimethylpropane-1,3-diyl)-bis (nitrilomethylidyne)]diphenolato}-palladium(II) ethanol hemisolvate (Wan Nazihah Wan Ibrahim *et al.*, 2008) in terms of the geometry around the central palladium atom.

In the title molecule (Fig. 1), the palladium atom lies on an inversion center and is coordinated to two ligand molecules through the oxygen and nitrogen atoms in a bidentate manner to form a perfect square planar geometry. The bond distances and bond angles in the title complex are normal (Allen *et al.*, 2002). The Pd1—O and Pd1—N bond lengths of 1.981 (2) and 2.039 (2)Å, respectively, in a square planar geometry are typical of square planar Pd(II) of Schiff bases (Adrian *et al.*, 2008). The dihedral angle between the benzene rings is 76.5 (2)°. The molecule is stabilized by C—H..π (C8—H8B···*Cg*3, C8—H8C···*Cg*4 and C13H-13 A···*Cg*3) interactions (Table 1).

### Experimental

The ligand, 2-hydroxyacetophenonebenzylimine, (1.1271 g, 5 mmol) was dissolved in hot ethanol (20 ml) in a round-bottomed flask. Palladium(II) acetate (0.5618 g, 2.5 mmol) was dissolved separately in hot ethanol (40 ml) and added into the flask containing the ligand solution. The mixture was stirred and refluxed for 5 h upon which green precipitate was formed. It was isolated by gravity filtration, washed with cold ethanol and air dried at room temperature. The solid product was recrystallized from chloroform yielding yellow crystals. Yield 87.80%; m.p. 529–530 K.

### Refinement

The H atoms were positioned geometrically with C—H = 0.97, 96 and 0.93 Å for methyl, methylene and aromatic groups, respectively, and constrained to ride on their parent atoms with *U*_iso_(H) = 1.5(methyl) or 1.2(methylene and aromatic) × *U*_eq_(C). The highest peak and deepest hole are located at 0.90 Å from Pd1 atom.

### Crystal data

**Table:**

[Pd(C_15_H_14_NO)_2_]	*F*(000) = 568
*M**_r_* = 554.94	*D*_x_ = 1.554 Mg m^−^^3^
Monoclinic, *P*2_1_/*n*	Mo *K*α radiation, λ = 0.71073 Å
Hall symbol: -P 2yn	Cell parameters from 4170 reflections
*a* = 11.188 (2) Å	θ = 2.1–25.5°
*b* = 9.4460 (17) Å	µ = 0.81 mm^−^^1^
*c* = 11.984 (2) Å	*T* = 298 K
β = 110.558 (4)°	Block, yellow
*V* = 1185.8 (4) Å^3^	0.28 × 0.20 × 0.12 mm
*Z* = 2	

### Data collection

**Table:**

Bruker SMART APEX CCD area-detector diffractometer	2177 independent reflections
Radiation source: fine-focus sealed tube	1939 reflections with *I* > 2/s(*I*)
graphite	*R*_int_ = 0.038
Detector resolution: 83.66 pixels mm^-1^	θ_max_ = 25.5°, θ_min_ = 2.1°
ω scan	*h* = −13→13
Absorption correction: multi-scan (*SADABS*; Bruker, 2000)	*k* = −11→11
*T*_min_ = 0.804, *T*_max_ = 0.908	*l* = −14→11
6420 measured reflections	

### Refinement

**Table:**

Refinement on *F*^2^	Primary atom site location: structure-invariant direct methods
Least-squares matrix: full	Secondary atom site location: difference Fourier map
*R*[*F*^2^ > 2σ(*F*^2^)] = 0.036	Hydrogen site location: inferred from neighbouring sites
*wR*(*F*^2^) = 0.109	H-atom parameters constrained
*S* = 1.18	*w* = 1/[σ^2^(*F*_o_^2^) + (0.0502*P*)^2^ + 0.7043*P*] where *P* = (*F*_o_^2^ + 2*F*_c_^2^)/3
2177 reflections	(Δ/σ)_max_ < 0.001
161 parameters	Δρ_max_ = 0.88 e Å^−^^3^
0 restraints	Δρ_min_ = −0.98 e Å^−^^3^

### Special details

**Table:**

Experimental. Analytical calculation for C_30_H_28_N_2_O_2_Pd: C,64.93; H,5.09; N,5.05. Found: C,64.58; H,4.97; N,4.94. IR (cm-1): v(C=N) 1586, v(C—O) 1323, v(C—H) 2977, v(C—N) 1355, v(Pd—O) 695, v(Pd—N) 476.
Geometry. All e.s.d.'s (except the e.s.d. in the dihedral angle between two l.s. planes) are estimated using the full covariance matrix. The cell e.s.d.'s are taken into account individually in the estimation of e.s.d.'s in distances, angles and torsion angles; correlations between e.s.d.'s in cell parameters are only used when they are defined by crystal symmetry. An approximate (isotropic) treatment of cell e.s.d.'s is used for estimating e.s.d.'s involving l.s. planes.
Refinement. Refinement of *F*^2^ against ALL reflections. The weighted *R*-factor *wR* and goodness of fit *S* are based on *F*^2^, conventional *R*-factors *R* are based on *F*, with *F* set to zero for negative *F*^2^. The threshold expression of *F*^2^ > σ(*F*^2^) is used only for calculating *R*-factors(gt) *etc*. and is not relevant to the choice of reflections for refinement. *R*-factors based on *F*^2^ are statistically about twice as large as those based on *F*, and *R*- factors based on ALL data will be even larger.

### Fractional atomic coordinates and isotropic or equivalent isotropic displacement parameters (Å^2^)

**Table:**

	*x*	*y*	*z*	*U*_iso_*/*U*_eq_	
Pd1	0.5000	0.5000	0.0000	0.02879 (16)	
O1	0.4462 (2)	0.5912 (3)	0.1236 (2)	0.0409 (6)	
N1	0.6541 (2)	0.6317 (3)	0.0459 (2)	0.0323 (6)	
C1	0.5319 (3)	0.6226 (3)	0.2285 (3)	0.0335 (7)	
C2	0.4944 (3)	0.6076 (4)	0.3284 (3)	0.0422 (8)	
H2A	0.4128	0.5748	0.3178	0.051*	
C3	0.5765 (4)	0.6406 (4)	0.4419 (3)	0.0468 (9)	
H3A	0.5503	0.6291	0.5069	0.056*	
C4	0.6971 (4)	0.6906 (4)	0.4583 (4)	0.0498 (10)	
H4A	0.7529	0.7116	0.5346	0.060*	
C5	0.7346 (3)	0.7095 (4)	0.3632 (4)	0.0428 (9)	
H5A	0.8156	0.7458	0.3760	0.051*	
C6	0.6551 (3)	0.6758 (3)	0.2454 (3)	0.0326 (7)	
C7	0.7007 (3)	0.6991 (3)	0.1464 (3)	0.0335 (7)	
C8	0.8047 (4)	0.8088 (4)	0.1658 (4)	0.0437 (9)	
H8A	0.7961	0.8529	0.0912	0.066*	
H8B	0.8867	0.7638	0.1977	0.066*	
H8C	0.7974	0.8793	0.2208	0.066*	
C9	0.7070 (3)	0.6577 (3)	−0.0483 (3)	0.0347 (7)	
H9A	0.6993	0.5717	−0.0945	0.042*	
H9B	0.7972	0.6790	−0.0114	0.042*	
C10	0.6432 (3)	0.7774 (3)	−0.1320 (3)	0.0321 (7)	
C11	0.5685 (3)	0.8786 (4)	−0.1050 (3)	0.0423 (8)	
H11A	0.5541	0.8737	−0.0333	0.051*	
C12	0.5149 (5)	0.9877 (4)	−0.1846 (5)	0.0554 (12)	
H12A	0.4646	1.0553	−0.1657	0.066*	
C13	0.5354 (6)	0.9965 (4)	−0.2906 (5)	0.0569 (13)	
H13A	0.4998	1.0701	−0.3432	0.068*	
C14	0.6090 (4)	0.8957 (4)	−0.3186 (4)	0.0531 (10)	
H14A	0.6231	0.9007	−0.3905	0.064*	
C15	0.6619 (3)	0.7873 (4)	−0.2398 (3)	0.0438 (9)	
H15A	0.7112	0.7193	−0.2595	0.053*	

### Atomic displacement parameters (Å^2^)

**Table:**

	*U*^11^	*U*^22^	*U*^33^	*U*^12^	*U*^13^	*U*^23^
Pd1	0.0270 (2)	0.0261 (2)	0.0302 (3)	−0.00145 (11)	0.00621 (17)	0.00023 (12)
O1	0.0325 (12)	0.0481 (14)	0.0402 (14)	−0.0030 (10)	0.0104 (11)	−0.0108 (11)
N1	0.0297 (13)	0.0273 (13)	0.0396 (16)	0.0003 (11)	0.0116 (12)	0.0024 (12)
C1	0.0351 (16)	0.0236 (15)	0.039 (2)	0.0035 (13)	0.0098 (15)	−0.0020 (14)
C2	0.0437 (19)	0.0346 (18)	0.053 (2)	−0.0016 (15)	0.0227 (18)	−0.0025 (16)
C3	0.057 (2)	0.049 (2)	0.036 (2)	0.0019 (18)	0.0177 (18)	−0.0037 (17)
C4	0.053 (2)	0.054 (2)	0.034 (2)	0.0033 (18)	0.0051 (18)	−0.0084 (18)
C5	0.0366 (18)	0.041 (2)	0.046 (2)	0.0004 (15)	0.0083 (17)	−0.0055 (17)
C6	0.0357 (16)	0.0268 (16)	0.0317 (18)	0.0023 (13)	0.0074 (14)	−0.0005 (13)
C7	0.0301 (16)	0.0257 (15)	0.038 (2)	0.0009 (13)	0.0041 (14)	0.0009 (14)
C8	0.0424 (19)	0.0353 (19)	0.050 (2)	−0.0090 (15)	0.0115 (18)	−0.0001 (17)
C9	0.0300 (16)	0.0345 (17)	0.040 (2)	−0.0021 (13)	0.0133 (15)	−0.0002 (14)
C10	0.0320 (16)	0.0330 (16)	0.0305 (18)	−0.0065 (13)	0.0099 (14)	−0.0012 (14)
C11	0.053 (2)	0.043 (2)	0.0287 (19)	0.0076 (16)	0.0111 (16)	0.0007 (16)
C12	0.074 (3)	0.040 (2)	0.052 (3)	0.0167 (18)	0.021 (3)	0.0026 (17)
C13	0.076 (4)	0.038 (2)	0.051 (3)	0.0001 (17)	0.016 (3)	0.0121 (16)
C14	0.072 (3)	0.051 (2)	0.043 (2)	−0.010 (2)	0.029 (2)	0.0042 (18)
C15	0.050 (2)	0.043 (2)	0.046 (2)	−0.0010 (16)	0.0268 (18)	−0.0005 (17)

### Geometric parameters (Å, °)

**Table:**

Pd1—O1	1.981 (2)	C7—C8	1.514 (4)
Pd1—O1^i^	1.981 (2)	C8—H8A	0.9600
Pd1—N1^i^	2.039 (3)	C8—H8B	0.9600
Pd1—N1	2.039 (3)	C8—H8C	0.9600
O1—C1	1.319 (4)	C9—C10	1.514 (5)
N1—C7	1.299 (4)	C9—H9A	0.9700
N1—C9	1.468 (4)	C9—H9B	0.9700
C1—C2	1.407 (5)	C10—C11	1.380 (5)
C1—C6	1.414 (4)	C10—C15	1.382 (5)
C2—C3	1.384 (5)	C11—C12	1.389 (6)
C2—H2A	0.9300	C11—H11A	0.9300
C3—C4	1.377 (5)	C12—C13	1.371 (8)
C3—H3A	0.9300	C12—H12A	0.9300
C4—C5	1.357 (6)	C13—C14	1.375 (6)
C4—H4A	0.9300	C13—H13A	0.9300
C5—C6	1.415 (5)	C14—C15	1.377 (6)
C5—H5A	0.9300	C14—H14A	0.9300
C6—C7	1.463 (5)	C15—H15A	0.9300
			
O1—Pd1—O1^i^	180.00 (8)	C7—C8—H8A	109.5
O1—Pd1—N1^i^	90.97 (10)	C7—C8—H8B	109.5
O1^i^—Pd1—N1^i^	89.03 (10)	H8A—C8—H8B	109.5
O1—Pd1—N1	89.03 (10)	C7—C8—H8C	109.5
O1^i^—Pd1—N1	90.97 (10)	H8A—C8—H8C	109.5
N1^i^—Pd1—N1	180.00 (11)	H8B—C8—H8C	109.5
C1—O1—Pd1	120.0 (2)	N1—C9—C10	114.3 (3)
C7—N1—C9	119.5 (3)	N1—C9—H9A	108.7
C7—N1—Pd1	125.1 (2)	C10—C9—H9A	108.7
C9—N1—Pd1	115.2 (2)	N1—C9—H9B	108.7
O1—C1—C2	117.0 (3)	C10—C9—H9B	108.7
O1—C1—C6	124.2 (3)	H9A—C9—H9B	107.6
C2—C1—C6	118.7 (3)	C11—C10—C15	118.3 (3)
C3—C2—C1	121.4 (3)	C11—C10—C9	123.0 (3)
C3—C2—H2A	119.3	C15—C10—C9	118.6 (3)
C1—C2—H2A	119.3	C10—C11—C12	120.3 (4)
C4—C3—C2	119.7 (3)	C10—C11—H11A	119.9
C4—C3—H3A	120.1	C12—C11—H11A	119.9
C2—C3—H3A	120.1	C13—C12—C11	120.5 (4)
C5—C4—C3	120.1 (4)	C13—C12—H12A	119.7
C5—C4—H4A	119.9	C11—C12—H12A	119.7
C3—C4—H4A	119.9	C12—C13—C14	119.6 (4)
C4—C5—C6	122.4 (4)	C12—C13—H13A	120.2
C4—C5—H5A	118.8	C14—C13—H13A	120.2
C6—C5—H5A	118.8	C13—C14—C15	119.8 (4)
C1—C6—C5	117.6 (3)	C13—C14—H14A	120.1
C1—C6—C7	122.5 (3)	C15—C14—H14A	120.1
C5—C6—C7	119.9 (3)	C14—C15—C10	121.4 (3)
N1—C7—C6	122.5 (3)	C14—C15—H15A	119.3
N1—C7—C8	120.9 (3)	C10—C15—H15A	119.3
C6—C7—C8	116.5 (3)		
			
O1^i^—Pd1—O1—C1	−151 (100)	C4—C5—C6—C7	179.6 (3)
N1^i^—Pd1—O1—C1	138.5 (2)	C9—N1—C7—C6	−178.3 (3)
N1—Pd1—O1—C1	−41.5 (2)	Pd1—N1—C7—C6	6.9 (4)
O1—Pd1—N1—C7	20.0 (3)	C9—N1—C7—C8	2.7 (4)
O1^i^—Pd1—N1—C7	−160.0 (3)	Pd1—N1—C7—C8	−172.1 (2)
N1^i^—Pd1—N1—C7	156 (100)	C1—C6—C7—N1	−24.1 (5)
O1—Pd1—N1—C9	−155.0 (2)	C5—C6—C7—N1	157.2 (3)
O1^i^—Pd1—N1—C9	25.0 (2)	C1—C6—C7—C8	155.0 (3)
N1^i^—Pd1—N1—C9	−19 (100)	C5—C6—C7—C8	−23.7 (5)
Pd1—O1—C1—C2	−144.1 (2)	C7—N1—C9—C10	−89.1 (3)
Pd1—O1—C1—C6	38.9 (4)	Pd1—N1—C9—C10	86.1 (3)
O1—C1—C2—C3	−178.7 (3)	N1—C9—C10—C11	17.5 (4)
C6—C1—C2—C3	−1.5 (5)	N1—C9—C10—C15	−163.5 (3)
C1—C2—C3—C4	0.7 (6)	C15—C10—C11—C12	−0.5 (6)
C2—C3—C4—C5	1.0 (6)	C9—C10—C11—C12	178.6 (4)
C3—C4—C5—C6	−1.8 (6)	C10—C11—C12—C13	−0.1 (7)
O1—C1—C6—C5	177.7 (3)	C11—C12—C13—C14	0.5 (8)
C2—C1—C6—C5	0.7 (4)	C12—C13—C14—C15	−0.3 (7)
O1—C1—C6—C7	−1.0 (5)	C13—C14—C15—C10	−0.3 (6)
C2—C1—C6—C7	−178.0 (3)	C11—C10—C15—C14	0.7 (5)
C4—C5—C6—C1	0.9 (5)	C9—C10—C15—C14	−178.4 (3)

Symmetry codes: (i) −*x*+1, −*y*+1, −*z*.

### Hydrogen-bond geometry (Å, °)

**Table:**

Cg3 and Cg4 are the centroids of the ???? and ???? rings, respectively.

**Table:**

*D*—H···*A*	*D*—H	H···*A*	*D*···*A*	*D*—H···*A*
C9—H9A···O1^i^	0.97	2.18	2.862 (4)	127
C11—H11A···N1	0.93	2.58	2.900 (5)	101
C8—H8B···Cg4^ii^	0.96	2.57	3.523 (5)	172
C13—H13A···Cg3^iii^	0.93	2.87	3.626 (5)	139

Symmetry codes: (i) −*x*+1, −*y*+1, −*z*;  (ii) *x*−1/2, −*y*+1/2, *z*−1/2; (iii) −*x*+1, −*y*+2, −*z*.
